# Multiplex Immunoassay Platforms Based on Shape-Coded Poly(ethylene glycol) Hydrogel Microparticles Incorporating Acrylic Acid

**DOI:** 10.3390/s120608426

**Published:** 2012-06-20

**Authors:** Saemi Park, Hyun Jong Lee, Won-Gun Koh

**Affiliations:** Department of Chemical and Biomolecular Engineering, Yonsei University, 134 Sinchon-Dong, Seodaemoon-Gu, Seoul 120-749, Korea; E-Mails: saemi.p@gmail.com (S.P.); brave520@hanmail.net (H.J.L.)

**Keywords:** suspension protein microarray, shape-coded, hydrogel microparticle, photopatterning, multiplex immunoassay

## Abstract

A suspension protein microarray was developed using shape-coded poly(ethylene glycol) (PEG) hydrogel microparticles for potential applications in multiplex and high-throughput immunoassays. A simple photopatterning process produced various shapes of hydrogel micropatterns that were weakly bound to poly(dimethylsiloxane) (PDMS)-coated substrates. These micropatterns were easily detached from substrates during the washing process and were collected as non-spherical microparticles. Acrylic acids were incorporated into hydrogels, which could covalently immobilize proteins onto their surfaces due to the presence of carboxyl groups. The amount of immobilized protein increased with the amount of acrylic acid due to more available carboxyl groups. Saturation was reached at 25% v/v of acrylic acid. Immunoassays with IgG and IgM immobilized onto hydrogel microparticles were successfully performed with a linear concentration range from 0 to 500 ng/mL of anti-IgG and anti-IgM, respectively. Finally, a mixture of two different shapes of hydrogel microparticles immobilizing IgG (circle) and IgM (square) was prepared and it was demonstrated that simultaneous detection of two different target proteins was possible without cross-talk using same fluorescence indicator because each immunoassay was easily identified by the shapes of hydrogel microparticles.

## 1. Introduction

Strong demands for achieving high-throughput and high-content protein analysis have brought into focus protein microarrays that are able to immobilize a large number of proteins simultaneously in a high-density format to identify protein-protein, protein-DNA, or protein-small molecule interactions [1,2]. A number of proteins can be immobilized either on a planar surface (positional or planar array) or on microparticles (suspension or solution array) to realize multiplexed and high-throughput protein microassays [3–6]. Positional arrays have been generated by the immobilization of proteins on different microdomains within a plane surface, such as a glass slide. Each protein is identified by x and y coordinates on the microarray [7–9]. Although a variety of positional microarrays have been developed and widely used for disease diagnosis, prognosis, biochemical analysis, and therapeutic regimes, non-positional arrays are becoming popular as an alternative approach for high-throughput assays [10–12]. These arrays are believed to have greater flexibility for multiplex assays and faster reaction kinetics. Suspension arrays utilize self-encoded particles as array elements instead of positionally-encoded spots on a planar surface. Currently, most suspension array systems employ optically-coded spherical microparticles to achieve multiplexed protein-based assays [13–17]. However, there are several disadvantages to using multiple colors as a means of encoding, including the limited number of color combinations and spectral overlap between an encoding color and the analyte-detection color.

Since the Doyle group developed outstanding methods for generating various shapes of hydrogel microparticles using continuous flow or stop-flow lithography [18,19], several groups have proposed using graphically or shape-coded hydrogel microparticles as a suspension microarray format for multiplexed bioassays [20–22]. Hydrogels are 3-dimensionally cross-linked polymers that can absorb water or other biological fluids and become swollen. They are an attractive support material for protein immobilization because the soft and hydrated environment of swollen hydrogels can provide proteins with near-physiological conditions that minimize denaturation and allow them to carry out their full biological functions. Among the various hydrogels, PEG-based hydrogels have been widely used in the biomedical field for many years because of their high water content, hydrophilicity, and biocompatibility. Lithographically-fabricated PEG hydrogel microparticles have been utilized for several biosensing systems based on enzyme-substrate reactions, DNA hybridization, and immunoassay [23,24]. In those applications, probe biomolecules are physically immobilized within a PEG hydrogel matrix because the PEG hydrogel has no available functional group for covalent immobilization. This strategy works very well for detecting small target molecules such as glucose, alcohol, and DNA. However, it might not be easy to detect proteins using immunoassay because most proteins are larger than the mesh sizes of PEG hydrogels. This issue could be alleviated if porogen is used to increase the porosity of hydrogels [25,26]. One strategy for overcoming these obstacles is to immobilize proteins on the outer surfaces of hydrogels so that target proteins can easily bind to probe proteins without the necessity to penetrate through hydrogel matrix. However, because of the unavailability of functional groups that can covalently immobilize proteins and non-adhesiveness toward proteins of PEG hydrogel, it is impossible to directly immobilize proteins onto the surface of bare PEG hydrogel.

In this study, we prepared a shape-coded suspension protein microarray capable of multiplexed immunoassays using PEG-based hydrogel microparticles. A photopatterning process fabricated different shapes of hydrogel microparticle. Acrylic acid was incorporated into the hydrogel to provide functional groups that can covalently immobilize probe proteins onto hydrogel surfaces. After confirming that IgG or IgM immobilized onto different hydrogel microparticles could each function in the immunoassay, the possibility of using shape-coded hydrogel microparticles for multiplexed immunoassay was demonstrated by simultaneously detecting two different analytes.

## 2. Experimental Section

### 2.1. Materials

Poly(ethylene glycol) diacrylate (PEG–DA) (M_W_ 575), 2-hydroxy-2-methylpropiophenone (HOMPP) photoinitiator, bovine serum albumin conjugated with fluorescein isocyanate (FITC-BSA), 1-ethyl-3(3-dimethylaminoprophyl)carbodiimide (EDC), *N*-hydroxysuccinimide (NHS), and acrylic acid were purchased from Sigma-Aldrich (Milwaukee, WI, USA). Poly(dimethylsiloxane) (PDMS) elastomer was purchased as Dow Corning Sylgard 184 (Midland, MI, USA) and is composed of prepolymer and a curing agent. FITC-rabbit anti-mouse immunoglobulin G(FITC-anti-IgG), mouse IgG, FITC-rabbit anti-mouse immunoglobulin M(FITC-anti-IgM), and mouse IgM were purchased from ZYMED Laboratories (San Francisco, CA, USA). Chrome soda lime photomask for the photolithographic patterning of hydrogels was purchased from Advanced Reproductions (Andover, MA, USA). Phosphate buffered saline (PBS, 0.1 M, pH 7.4) consisted of 1.1 mM potassium phosphate monobasic, 3 mM sodium phosphate dibasic heptahydrate, and 0.15 M NaCl in deionized water.

### 2.2. Preparation of Hydrogel Microparticles

Hydrogel microparticles were prepared by UV-initiated free radical polymerization as described in our previous study [27]. The initial gel precursor solution was composed of 50% v/v PEG-DA (MW 575) and 2% v/v HOMPP as photoinitiator in PBS solution. A 50 μL aliquot of precursor solution was dropped onto a glass slide coated with cured PDMS and was spread by covering it with a PDMS-coated photomask. The precursor solution was then exposed to 365 nm, 300 mW/cm^2^ UV light (EFOS Ultracure 100ss Plus, UV spot lamp, Mississauga, ON, Canada) for 1 s through the photomask. UV photopolymerization created hydrogel micropatterns because only precursor solution exposed to UV light underwent free-radical cross-linking and became insoluble in common PEG solvents such as water. The final hydrogel microparticles were obtained by flushing the PDMS-coated glass slide and photomask and collecting the released hydrogel micropatterns. To incorporate carboxyl groups into hydrogel, a given amount of acrylic acid was added to the precursor solution and copolymerized with PEG-DA. Resultant hydrogel microparticles were washed overnight to remove any unreacted or unbound molecules from the hydrogels.

### 2.3. Immobilization of Proteins onto Hydrogel Microparticles

Proteins such as FITC-BSA, IgG, and IgM were covalently immobilized on hydrogel microparticles via an EDC/NHS-mediated reaction. Carboxylated hydrogel microparticles were immersed into a solution containing 2 mM EDC and 5 mM NHS for three hours to convert the carboxyl groups in acrylic acid into reactive intermediates susceptible to attack by amine groups in proteins. The activated hydrogel microparticles were then washed with PBS and immediately reacted with protein solutions (20 μg/mL for IgG and IgM, 1.0 mg/mL for FITC-BSA) for three hours at 4 °C. The reactions resulted in amide bond formation that covalently attached proteins to the hydrogel surface. After immobilization of proteins, hydrogel microparticles were thoroughly washed with PBS to remove weakly-bound or physically-entrapped proteins.

### 2.4. Immunoassay with Hydrogel Microparticles

For immunoassays, IgG or IgM-immobilized hydrogel microparticles were incubated with blocking solution (1 wt% BSA in PBS solution) for two hours and subsequently reacted with different concentration of FITC-anti-IgG or FITC-anti-IgM at 4 °C for two hours. After the reaction, hydrogel microparticles were thoroughly washed to remove physically-adsorbed or hydrogel-entrapped anti-IgG or anti-IgM. Immunoreactions on the hydrogel microparticles were visualized and quantified using fluorescence microscopy. To investigate cross-reactivity, IgG-immobilized hydrogel microparticles were reacted with different concentration of anti-IgM, or IgM-immobilized hydrogel microparticles were reacted with anti-IgG. A Zeiss Axiovert 200 microscope equipped with an integrated color CCD camera (Carl Zeiss Inc., Thornwood, NY, USA) was used to obtain fluorescent and optical images. The fluorescence intensity was quantified using commercially available image analysis software (KS 300, Carl Zeiss Inc.).

## 3. Results and Discussion

**Figure 1.**
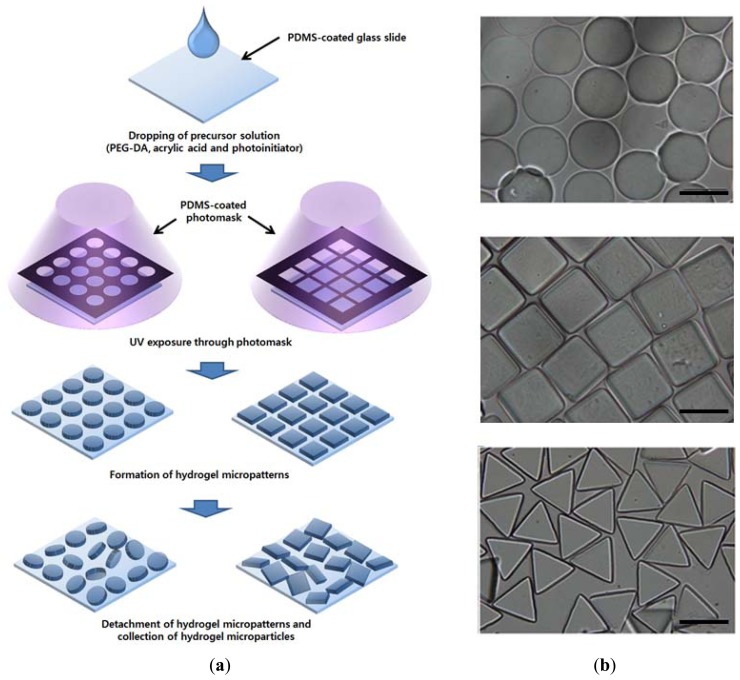
Fabrication of hydrogel microparticles using photopatterning. (**a**) Schematic illustration of preparing hydrogel microparticles. (**b**) Optical images of resultant hydrogel microparticles with different shapes (scale bar: 200 μm).

A photopatterning process, described in Figure 1(a), was used to prepare suspension arrays of the hydrogel microparticles. The precursor solution could cross-link to form a gel under UV light, a characteristic that was used to create negative patterns by photolithography. Light projected through the photomask created polymerized regions that corresponded to the photomask pattern. In this study, glass slides and photomasks were coated with a 20-μm-thick layer of hydrophobic PDMS to suppress their attraction to the hydrophilic hydrogel micropatterns. This process facilitated removal of hydrogel micropatterns from the substrates. Finally, the desired hydrogel microparticles were obtained by collecting detached hydrogel micropatterns, as shown in Figure 1(b). Although PEG hydrogel microparticles of various sizes and shapes could be fabricated by simply changing the photomask design [22], we used photomasks containing arrays of circles, triangles and squares with a lateral dimension of 100 or 200 μm. Due to protein-repelling behavior and unavailable functional groups, proteins cannot immobilize onto the PEG hydrogel surface via physical adsorption or covalent linkage. For covalent immobilization of proteins, a various amount of acrylic acid was copolymerized with PEG-DA. Using the EDC/NHS-mediated reaction, carboxyl groups in acrylic acids were converted to N-hydroxysuccinimide ester, which can react with amine groups in proteins to form a stable amide linkage.

**Figure 2.**
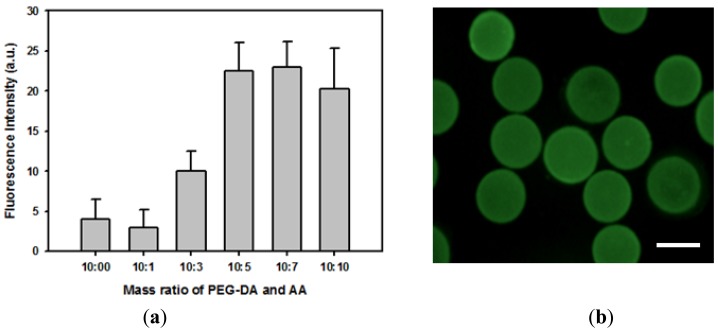
Immobilization of FITC-BSA onto hydrogel microparticles. (**a**) Effect of acrylic acid contents on the relative amount of FITC-BSA immobilized onto hydrogel microparticles. (**b**) Fluorescence image of hydrogel microparticles that were incubated with FITC-BSA (weight ratio of PEG-DA and acrylic acid was fixed to 2:1) (scale bar: 200 μm).

The feasibility of covalently immobilizing proteins onto hydrogel microparticles was investigated by incubating FITC-BSA with activated carboxylated hydrogel microparticles. Relative amounts of immobilized proteins were determined from the quantitative fluorescence intensity data. Figure 2(a) indicates that almost no protein was physically adsorbed onto bare PEG hydrogel microparticles. In addition, more proteins could be covalently immobilized onto hydrogel microparticles containing a higher concentration of acrylic acid due to a greater number of available carboxyl groups. The amount of immobilized protein reached maximum value when the volume ratio of PEG-DA and acrylic acid was 2:1. Adding acrylic acid above this concentration did not further increase the protein-loading density of hydrogel microparticles. Therefore, hydrogel microparticles made from a precursor solution containing 50% v/v PEG-DA, 25% v/v acrylic acid, and 2% v/v HOMPP was used throughout this study. Figure 2(b) shows the fluorescence image of hydrogel microparticles immobilizing FITC-BSA, demonstrating that protein was homogeneously distributed through hydrogel microparticles. Covalent immobilization caused more denaturation of proteins than physical immobilization methods such as physical adsorption and physical entrapment. However, in case of physical adsorption, the amount of proteins that could be immobilized onto hydrogel surface were very low, while physical entrapment suffered from diffusion limitation of target molecules, especially large molecules such as proteins. Therefore, we utilized covalent immobilization in this study.

**Figure 3.**
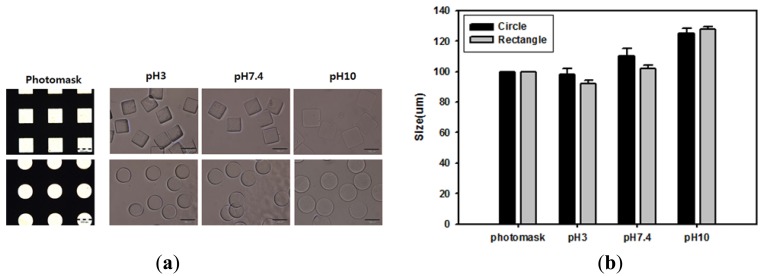
Effect of pH on the morphology of hydrogel microparticles. (**a**) Optical images of hydrogel microparticles that were immersed in different pH buffer solution for 2 h (scale bar: 100 μm). (**b**) Actual lateral dimensions of hydrogel microparticles in different pH buffer solutions.

Acrylic acid-incorporating hydrogel can achieve different levels of swelling at different pH levels by ionizing carboxyl groups above their pKa (4.7). Because of this, changing the pH was expected to alter the morphology of carboxylated hydrogel microparticles. To investigate the pH-response of hydrogel microparticles, acrylic acid-incorporating hydrogel microparticles were fabricated using a photomask containing arrays of circles or squares with a lateral dimension of 100 μm. The microparticles were then incubated with different pH buffer solutions. As shown in the optical images of Figure 3(a) and the quantitative data of Figure 3(b), hydrogel microparticle sizes increased as the pH of the buffer solution increased. This change occurred because acrylic acid groups in the hydrogel became charged and induced hydrogel swelling under conditions over the pKa value threshold. However, hydrogel microparticles maintained their original shapes and the size increase was less than 10% at pH 7.4, the value at which our immunoassay was performed.

**Figure 4.**
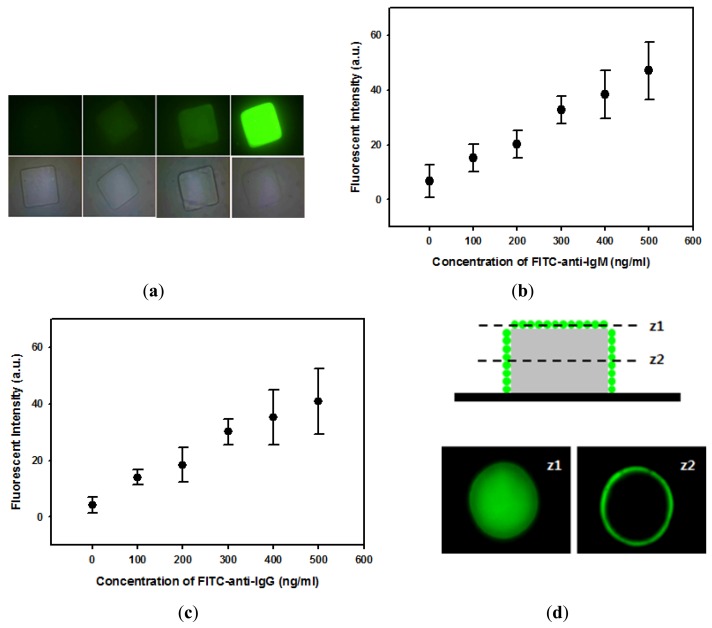
Immunoassays using hydrogel microparticles immobilizing IgG or IgM. (**a**) Optical and fluorescence images of IgM-immobilized hydrogel microparticle that reacted with different concentration of FITC-labeled anti-IgM. (**b**) Relationship between concentration of anti-IgM and the fluorescence intensity. (**c**) Relationship between concentration of anti-IgG and the fluorescence intensity. (**d**) Confocal slice images at different z position (z1, z2).

After successful covalent immobilization of proteins onto hydrogel microparticles, immunoassays were performed to test the potential application of hydrogel microparticles as suspension array-based biosensors. First, IgM was immobilized onto square-shaped hydrogel microparticles and reacted with FITC-anti-IgM. Fluorescent images demonstrated that anti-IgM bound specifically to IgM-immobilized microparticles and that fluorescence intensity increased with anti-IgM concentration (Figure 4(a)). Figure 4(a) also indicates that anti-IgM bound almost homogeneously on hydrogel microparticles. Figure 4(b) quantitatively demonstrates that the fluorescence intensity linearly increased with concentrations of anti-IgM. IgG was also immobilized onto circular hydrogel microparticles to investigate the reaction between IgG and anti-IgG. Similarly with the IgM/anti-IgM reaction, the fluorescence intensity of hydrogel microparticles was linearly dependent on the concentration of IgG as shown in Figure 4(c). The locations of immunoassay binding events were further investigated with confocal microscopy. Figure 4(d) shows slice images of IgG immobilized hydrogel microparticles that reacted with FITC-anti-IgG, obtained at two different height positions (z). As shown in these images, most FITC-anti-IgG was bound to the sidewall and to the top of the hydrogel microstructure. This result indicates that the hydrogel mesh size was not large enough to permit complete diffusion of FITC-anti-IgG into the hydrogel microstructure. This resulted in FITC-anti-IgG binding only in the outer region of hydrogel microparticles, leaving empty space inside the hydrogel microparticles (Figure 4(d)).

**Figure 5.**
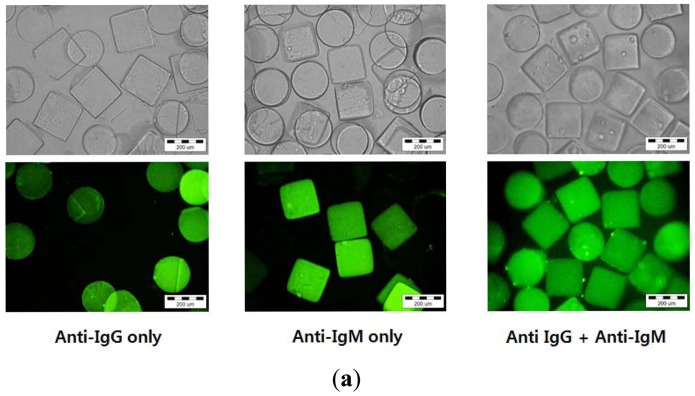

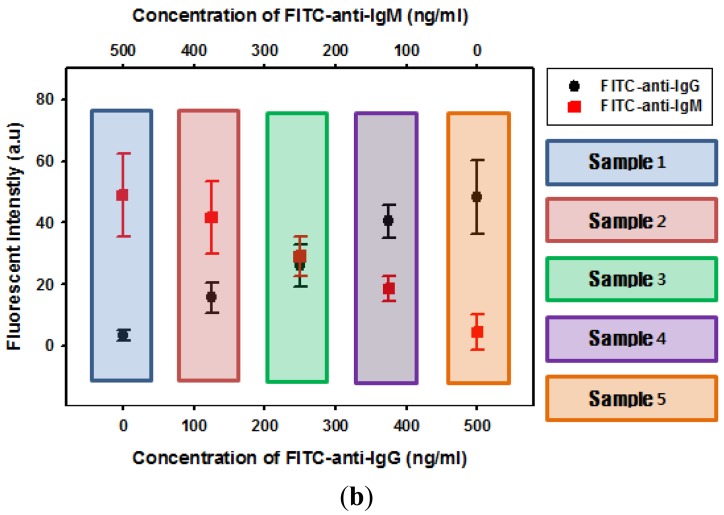
Multiplexed immunoassays using shape-coded hydrogel microparticles immobilizing IgG (circle) or IgM (square). (**a**) Optical and fluorescence image depending on sample composition. (**b**) Fluorescence intensity of circular and square hydrogel microparticles which were reacted with five different samples (Each sample was composed of anti-IgG and anti-IgM at different molar ratio).

Finally, we demonstrated simultaneous analysis of different immunoassays using suspension arrays of hydrogel microparticles. As a proof of concept, multiplex analysis was performed with two different shapes of hydrogel microparticles, circular hydrogel immobilizing IgG and square hydrogel immobilizing IgM. The two suspension arrays were prepared separately and combined in a well plate at a 1:1 volume ratio. Each reaction was identified by the hydrogel microparticle shape, generating a shape-coded suspension microarray system. Five different samples (mixtures of FITC-anti-IgG and FITC-anti-IgM) were prepared and reacted with the suspension arrays of shape-coded hydrogel microparticles. When a solution containing either anti-IgG or anti-IgG was introduced, only the hydrogel microparticles immobilizing corresponding probe molecules emitted green florescence without any cross-talk, as shown in Figure 5(a). Conversely, introducing a solution containing both anti-IgG and anti-IgM resulted in fluorescence from both hydrogel microparticle shapes (Figure 5(a)). Figure 5(b) shows the change in fluorescence intensity from circular and square microparticles when a mixture of anti-IgG and anti-IgM with different concentration ratios was reacted with microparticles. Both anti-IgG and anti-IgM were successfully detected. The intensity values from both shapes of hydrogel microparticles were in good agreement with previous results from anti-IgG-only and anti-IgM-only solutions. This indicated that the presence of one analyte does not have an effect on the other analyte.

## 4. Conclusions

This paper has described the preparation of hydrogel microparticles and their potential application to multiplexed immunoassays in a suspension array format. Hydrogel microparticles were fabricated by a simple photopatterning process that cross-linked liquid precursor solution containing acrylic acid, PEG-DA, and photoinitiator to form hydrogel. Because of the carboxyl groups in acrylic acid, resultant hydrogel microparticles were capable of covalently immobilizing proteins via an EDC/NHS-mediated reaction. For the immunoassay, a suspension array of hydrogel microparticles immobilizing IgM and IgG were prepared. The binding events between IgM and anti-IgM and between IgG and anti-IgG were successfully investigated using fluorescence detection. Furthermore, a mixture of two different shapes of hydrogel microparticle-immobilizing IgM and IgG was prepared. This shape-coded suspension array of hydrogel microparticles could simultaneously characterize two different immunoassays without cross-talk, despite using the same fluorescence indicator. Since many unique shapes of hydrogel microparticles can be prepared by changing photomask designs, more than two analytes can be detected by randomly assembling a mixture of desired hydrogel microparticles and incorporating appropriate receptor molecules.
